# Cancer diagnosis in the post-coronavirus disease era: correspondence

**DOI:** 10.1590/1806-9282.20240851

**Published:** 2024-09-16

**Authors:** Hinpetch Daungsupawong, Viroj Wiwanitkit

**Affiliations:** 1 Private Academic Consultant – Phonhong, Lao People's Democratic Republic.; 2 Saveetha University, Saveetha Institute of Medical and Technical Sciences, Saveetha Dental College and Hospitals, Department of Research Analytics – Chennai, India.

Dear Editor,

We would like to share ideas on the publication "Cancer diagnosis in the post-coronavirus disease era: the promising role of telepathology and artificial intelligence^1^." The major effects of the COVID-19 pandemic on cancer detection and treatment around the world are covered in this article. Due to delays in diagnosis and treatment brought on by the pandemic's decline in cancer diagnoses, the number of avoidable deaths from cancer of all kinds has increased. Artificial intelligence (AI) and telepathology are emphasized as viable means of addressing these issues by offering prompt and accurate diagnoses, particularly in isolated or resource-constrained locations. To guarantee the ethical and efficient application of these technologies in cancer detection and treatment, however, a number of regulatory and ethical issues need to be addressed.

The use of retrospective data from multiple studies to calculate the effect of the COVID-19 pandemic on cancer diagnosis and mortality is one of the article's points. Although these studies offer insightful information, differences in population demographics and data-gathering techniques may have an impact on the accuracy and dependability of data. To maximize their benefits and resolve any potential issues, it is also important to investigate the limitations of telepathology and AI technologies in cancer diagnosis, such as the requirement for qualified specialists and regulatory control.

How healthcare systems and legislators may successfully use telepathology and AI technology in cancer diagnostic and treatment procedures after COVID-19 is a major topic raised by this study. To guarantee cancer patients receive fair healthcare, it is crucial to take these technologies’ scalability, accessibility, and affordability into account. Furthermore, to maximize patient outcomes and treatment efficacy, it is important to carefully consider how healthcare practitioners might use these technologies as auxiliary tools while maintaining clinical judgment and experience.

Subsequent research paths in this field may concentrate on assessing the long-term effects on patient outcomes and survival rates of delayed cancer diagnoses during the COVID-19 epidemic. Furthermore, research on how well telepathology and AI technologies support cancer patients’ early detection, individualized treatment plans, and follow-up could yield important insights about how to better offer cancer care. To improve cancer detection and treatment outcomes in the post-pandemic age, collaboration between researchers, healthcare providers, and technology developers is required to further develop and refine these technologies.
